# OD12 Large Language Models As Powerful Tools In Health Technology Assessment

**DOI:** 10.1017/S0266462324001478

**Published:** 2025-01-07

**Authors:** Joan Segur-Ferrer, Carolina Moltó-Puigmartí, Berta Mestre Lleixà, Anna Godo Pla, Roland Pastells Peiró, Laura Llinàs-Mallol, Maria-Dolors Estrada Sabadell, Rosa Maria Vivanco-Hidalgo

## Abstract

**Introduction:**

In recent years, large language models (LLMs) have emerged as powerful tools in natural language processing, presenting diverse opportunities across various sectors. In this context, the Agency for Health Quality and Assessment of Catalonia (AQuAS) is actively investigating how LLMs can enhance the development of health technology assessment (HTA) reports.

**Methods:**

To assess the potential of LLMs in the development of HTA reports, our initial step involved a comprehensive review of technical literature to understand the functionalities of existing LLM tools. This effort was followed by a systematic identification of specific HTA report development tasks that these models could potentially facilitate. We then rigorously evaluated the performance of these tools in executing the delineated tasks, with a focus on meticulously recording the applied prompts and areas of high and low effectiveness, alongside any encountered errors and identified risks.

**Results:**

Among a range of commercial tools, ChatGPT and Microsoft Copilot were selected for exploration due to their accessibility, ease of use, and versatility. Both tools demonstrated acceptable performance in certain tasks (e.g., data extraction), while their efficacy was notably lower in others (e.g., information search). Additionally, a range of risks requiring mitigation were identified (e.g., hallucination or authority bias). Consequently, we drafted a proposal for an internal guide with directives for the technical staff of the HTA unit on utilizing these two tools. Additionally, we constituted a working group.

**Conclusions:**

LLMs have emerged as promising tools in the field of HTA. Over the past months, AQuAS has been investigating the potential of these models to improve the HTA report development process, targeting enhanced efficiency and improved quality. This exploration has led to the identification of numerous opportunities and associated risks within this innovative application.

